# Oral lichen planus and apremilast: Case series and literature review

**DOI:** 10.1016/j.jdcr.2026.05.071

**Published:** 2026-06-18

**Authors:** Jamie E. Ganem, Shannon Wessel, David Baltazar, Christina L. Harview, Margaret Kessler, Lindsay Ackerman

**Affiliations:** a Department of Internal Medicine, University of Arizona College of Medicine —Phoenix, Phoenix, Arizona; b Internal Medicine, University of Arizona College of Medicine — Phoenix, Phoenix, Arizona; c Medical Dermatology Specialists — Phoenix, Phoenix, Arizona

**Keywords:** apremilast, oral lichen planus, oral ulcers, phosphodiesterase-4 inhibitor, “swish-and-spit” apremilast

## Introduction

Apremilast is a phosphodiesterase-4 inhibitor with downstream anti-inflammatory effects that increases production of cyclic adenosine monophosphate and subsequently reduces interleukin (IL)- 2, IL-8, interferon-gamma, and tumor necrosis factor (TNF). It is currently FDA approved for the treatment of psoriasis, psoriatic arthritis, and oral ulcers in Behcet’s disease, and has increasingly been used off label for other inflammatory conditions, including lichen planus (LP).^1^

LP is believed to be a chronic, T-cell mediated inflammatory condition that can be idiopathic or associated with exposure to anti-malarials, beta-blockers, gold, and hepatitis C.^2^ LP can affect the skin and mucous membranes; when present on the oral mucosa, LP is re-named oral lichen planus (OLP). OLP tends to be more chronic, poorly responsive to therapy, and more debilitating for patients.^3^ Treatment mainly consists of topical steroids, though steroid rinse solutions or ointments have limited benefit, poor tolerance, and adverse effects (AEs) in the mouth (eg oral candidiasis). Therefore, OLP patients often require systemic immunosuppressive agents.^3^ However, immunosuppressive agents are frequently discontinued, even after short periods, due to AEs—highlighting the need for better therapies.^4^

Given the pathophysiology of OLP as an interferon gamma and TNF-mediated T-cell disease, apremilast presents a logical treatment choice and may prove more tolerable than prior treatments.^1^^,^^2^ Herein, we present 6 patients with OLP managed with either oral apremilast or “swish-and-spit” dissolved apremilast (one 30 mg tablet dissolved in 16.9 ounces of water, shaken prior to using 1-2 ounces) and summarize the results of our literature review.

## Methods

The electronic medical record at Medical Dermatology Specialists—Phoenix was searched for patients with OLP who were prescribed apremilast. These charts were reviewed to ensure relevance. Providers within the practice were also asked to recall any patients with OLP treated with any form of apremilast. For the literature search, 2 authors (JG and SW) conducted a literature search in PubMed using the search terms, “lichen planus” and “apremilast,” yielding 31 articles. Titles and abstracts were screened for relevance, and 9 articles were selected for full review, comprising 58 total patient cases (Supplementary Fig 1, available via Mendeley at https://data.mendeley.com/datasets/hjcxnj3bfz/1). From each included study, data was extracted on patient demographics (age, sex), prior failed therapies, apremilast dosing and administration route, treatment duration, clinical outcomes, and AEs. The institution librarian also searched on Embase with the query, “apremilast for oral lichen planus.” Articles that provide detailed patient information are represented in Table I.

**Table I:** Cases of oral lichen planus treated with apremilast with treatment course, outcomes, and side effects

Author (y)	Patient age	Sex	Apremilast trial	Results	Side effects
Paul et al (2012)^5^	Not reported	Female	20 mg BID × 12 wk	- Bilateral oral lesions improved from 40% to 12% involvement. - At day 15, PGAMD score was, “marked resolution” - At the end of the study, PGAMD score was, “moderate improvement.”	Right bundle branch block (not clinically significant)
Bettencourt (2016)^6^	73	Female	30 mg BID × 5 mo	- Mouth sores healed - Patient continued to take low dose Prednisone (5 mg daily) to prevent flares	None
Bettencourt (2016)^6^	71	Female	30 mg BID × 2 wks; 30 mg QD × 5.5 mo	- Good control and no oral discomfort - Only minimal erythema of the oral mucosa	Diarrhea, Nausea
Bettencourt (2016)^6^	66	Female	30 mg BID × 3 mo	- Mouth completely free of sores and no pain with eating	None
Hafner et al (2016)^7^	74	Female	20 mg BID × 4 wk	- Erosive stomatitis in complete remission - No clinical dysphagia	None
AbuHilal et al (2016)^8^	44	Female	30 mg BID × 10 d; 30 mg QD × 10 wk	- Marked improvement in both buccal and gingival lesions, along with significant reduction in pain and discomfort - Quality of life improved with easier speaking, eating and chewing	Nausea
Kim-Lim et al (2023)^9^	36	Female	Crush 60 mg dissolved in 500 mL water × 5 mo	- Complete resolution of lip lesions - Significant reduction in pain	None
Sil et al (2024)^10^	48	Female	30 mg BID × 12 wk	Pretreatment >12-wk Visit Scores - PGA: 4 > 1 - SGA: 5 > 0 - TALSS: 8 > 1	Mild gastric irritation
Sil et al (2024)^10^	59	Female	30 mg BID × 12 wk	Pretreatment >12-wk Visit Scores - PGA: 3 > 0 - SGA: 4 > 0 - TALSS: 7 > 1	Mild gastric irritation
Patient 1	83	Female	10 mg dissolved in 2-4 oz of water QHS × 1 mo	- Old lesions symptomatically reduced	New lesion on the tongue
Patient 2	64	Female	30 mg apremilast dissolved in 12 oz water + Acitretin 17.5 mg + Dexamethasone swish and spit + PRN Clotrimazole troche; 30 mg apremilast BID + 15 mL oral Tacrolimus PRN × 3 mo	- Significant improvement in both disease and pain	Nausea
Patient 3	72	Female	60 mg apremilast dissolved into 60 oz of water + Clobetasol 0.05% gel PRN × 6 mo	- Oral erosions reduced on exam with significant improvement in disease	Oral thrush
Patient 4	49	Female	30 mg BID × 9 mo	- Improvement in both Oral and Genital Lichen Planus	None
Patient 5	84	Female	30 mg BID + Lidocaine mouthwash + Mycophenolate Mofetil × 6 mo	- 60% improvement in oral lesions and less pain with eating	Hypersalivation
Patient 6	75	Female	30 mg BID × 4 mo	- 75% improvement in disease before discontinuation	Significant weight loss and discontinuation of solo therapy
			Upadacitinib with apremilast mouth wash	- Sustained disease improvement	None

The first 9 cases are those reported in the literature with extractable information. The last 6 rows represent the patients in our case series treated at Medical Dermatology Specialists—Phoenix.

## Results

### Case series

The 6 selected OLP patients are all female with an age range of 49-84. Two patients were treated with “swish-and-spit” dissolved apremilast, 2 patients were treated with oral apremilast 30 mg twice daily, and the remaining 2 patients received both oral and “swish-and-spit” during their treatment courses. In patients treated with both formulations, dissolved “swish-and-spit” apremilast was initiated first for disease confined to the oral mucosa to minimize systemic AEs; oral apremilast was introduced when response was incomplete, and in some cases was used alongside adjunctive therapies based on disease severity and prior treatment history. Patient characteristics and treatment details are summarized in Table I.

### Literature review

Paul et al (2012) was the first to describe apremilast’s use in OLP. Although only 1 patient had mucosal involvement, apremilast 20 mg twice daily resulted in improvement from 40% to 12% involvement of the bilateral buccal mucosa over 12 weeks. The Physician Global Assessment of Mucosal Disease (PGAMD) improved to “marked resolution” by day 15 and “moderate improvement” at study completion.^5^ A new incomplete right bundle branch block was noted but deemed clinically insignificant—treatment was continued.^5^

Bettencourt’s 2016 study includes 3 cases of refractory OLP treated with apremilast. The first case, a 73-year-old woman, presented with a 12-year history of painful oral erosions unresponsive to multiple systemic and topical therapies.^6^ After initiating apremilast 30 mg twice daily, pain and sores resolved within 2 weeks. Despite 2 flares, the patient continued the regimen for more than 5 months with satisfactory control of her OLP and no AEs.^6^

The second case involved a 71-year-old female with biopsy-confirmed OLP refractory to methylprednisolone, hydroxychloroquine, and topical steroids.^6^ Apremilast was initiated at 30 mg twice daily but was reduced to once daily due to nausea and diarrhea. Symptoms resolved within 4 weeks, and disease control was maintained for 6 months.^6^

The final case was a 66-year-old female with diagnosed OLP and eating discomfort despite triamcinolone paste. She achieved complete symptom resolution after 3 months of apremilast 30 mg twice daily.^6^

Hafner et al (2016) described a 74-year-old woman with OLP who initially developed genital skin changes. After erosive stomatitis and swallowing difficulties developed, LP was diagnosed. Methylprednisolone and azathioprine were both discontinued due to inefficacy and AEs respectively, and esophageal dilations were repeatedly ineffective. Following 4 weeks of apremilast, oral and esophageal symptoms resolved. Esophagoscopy confirmed recovery of the esophageal mucosa without recurrence of stenosis.^7^

AbuHilal et al documented a 44-year-old female with remissive cutaneous LP that presented with 5 years of progressive oral and gingival pain. Topical clobetasol and tacrolimus, intralesional triamcinolone, systemic prednisone, cyclosporine, acitretin, dapsone, hydroxychloroquine, azathioprine, and mycophenolate mofetil had all been utilized with no effect.^8^ Histopathological examination diagnosed OLP and desquamative gingivitis. Apremilast 30 mg twice daily was initiated and later reduced to once daily due to nausea. Following 12-weeks, marked improvement was observed in both buccal and gingival lesions, with significant improvement of quality of life.^8^

The abstract from Khan et al presented a retrospective analysis of 7 patients with LP treated with apremilast 30 mg twice daily after treatment failure. Three were treated simultaneously with topical agents. Six (85.7%) patients had symptomatic improvement, 2 (28.6%) had significant improvement, and 3 (42.9%) were clear. Four (57.1%) patients had no loss in response after treatment for more than a year. Three (42.9%) patients experienced mild side effects (headache, diarrhea, insomnia).^11^

Perschy et al analyzed 11 patients with OLP (6 females, mean age 62.4 years) from 3 European dermatology clinics after 6 and 12 weeks of apremilast use after prior treatment failure.^12^ Outcomes included the Physician Global Assessment (PGA) and Numeric Rating Scale (NRS). At week 12, 55% (6/11) of patients showed improvement in symptoms and clinical features—median PGA scores improved from 2 prior to treatment, to 1 after 12 weeks. Three patients experienced long-term benefits, with 2 continuing treatment after 11 months.^12^

Viswanath et al conducted a prospective, open-label study of 26 patients with biopsy-confirmed LP treated with apremilast 30 mg twice daily for 12 weeks. OLP and other LP subtypes were included.^13^ Efficacy was assessed using PGA, Subject Global Assessment (SGA), and Target Area Lesion Symptom Score (TALSS). PGA demonstrated that 34.61% (9/26) of patients showed greater than two-grade improvement and 34.61% had a one-grade improvement. 30.76% showed no improvement. SGA demonstrated that 42.3% of patients reported >50% improvement in lesions and 7.69% achieved >75% improvement, while 19.23% saw no improvement.^13^ Baseline TALSS was: 6.35 ± 1.64 and improved significantly by week 12: 3.88 ± 2.14 (*P* < .0001). Mild AEs were reported in 23.07% (6/26) of patients, however there were no serious AEs nor study discontinuations.^13^

Kim-Lim et al describes a 36-year-old woman with a 6-month history of painful OLP unresponsive to topical and systemic therapies who declined systemic immunosuppression.^9^ Crushed apremilast (60 mg dissolved in water for “swish and spit”) resulted in complete lesion resolution after 5 months, without side effects.^9^

More recently, Sil et al reported on a 48-year-old female, with a 20-year smokeless tobacco history, presenting with bilateral buccal mucosa burning refractory to corticosteroids.^10^ Incisional biopsy-confirmed OLP, and the patient was started on apremilast 30 mg twice daily for 12 weeks.

In the same report, the authors reported another case of a 59-year-old female, with a 15-year chewing tobacco history, presenting with 10 years of pain and left buccal mucosa burning also refractory to corticosteroids. After incisional biopsy-confirmed OLP with dysplasia, she too was started on apremilast 30 mg twice daily for 12 weeks.^10^

PGA, SGA, and TALSS were used for patient assessment at 6 and 12-week follow-up visits. At 6 weeks, there was a 2 grade or more reduction in all 3 scales in both patients. And 12 weeks, all 3 scales recorded a near zero-point reading, implying complete resolution in both patients.^10^ Apart from mild gastric irritation, no other AEs were noted.

## Discussion

This case series and literature review highlights the growing evidence for apremilast as an effective treatment for OLP, particularly in refractory cases. Our findings contribute additional real-world data demonstrating efficacy, tolerability, and durability of response.

Most patients across our case series had longstanding, biopsy-proven OLP resistant to standard therapies. The introduction of apremilast resulted in significant improvements in mucosal lesions, symptoms, and quality of life for most patients. These findings align with prior studies, as outlined in this review. Our findings further solidify apremilast's potential utility as a second-line or adjunctive therapy.

Furthermore, across multiple reports, apremilast provided clinical improvement within weeks of initiation. Several cases, such as those in the Sil et al and Hafner et al studies, showed complete resolution of oral lesions by 12 weeks, even with severe or erosive OLP. Sustained remission in long-term users underscores its durability, adding to the evidence from Viswanath et al, where 42.3% of patients with OLP and other non-mucosal subtypes achieved >50% improvement in symptom scores.^13^

Adverse events in our series were generally mild and manageable with dose modifications; only 1 patient discontinued therapy due to severe weight loss. Excluding this single patient, there were no AEs that required drug discontinuation, consistent with prior studies.^6^^,^^8^^,^^10^ Excluding the patient in our series, apremilast discontinuation only occurred in Perschy et al, of which 7 patients (63.6%) discontinued treatment—but only 3 (27.3%) from AEs and 4 (36.4%) due to disease progression. Overall, across the 64 cases between the literature review and our case series, only 8 patients (12.5%) discontinued apremilast, 4 (6.25%) due to AEs. This safety profile makes apremilast a viable option for patients contraindicated for systemic immunosuppressants. Of note, roflumilast, another phosphodiesterase-4 inhibitor, may represent a more accessible alternative, as it is often more readily available with fewer cost and insurance barriers in the United States. Although data is limited, its pharmacologic similarity warrants further investigation.^14^

By incorporating cases of erosive, refractory, and tobacco-associated OLP, this review addresses gaps in existing literature, which often focuses on single disease presentations. These findings suggest apremilast may have a role in both severe and atypical OLP cases, expanding its potential applications. Furthermore, the discussion on dose adjustments (eg, 30 mg twice daily to once daily) and alternative delivery methods offers practical guidance for clinicians, particularly for patients unwilling or unable to tolerate systemic therapy, supporting the findings in Kim-Lim et al.^9^ Reports of sustained remission highlight apremilast's potential for long-term disease control. Together, this adds a real-world dimension to the growing body of evidence supporting apremilast's efficacy.

An essential contribution to patient care by our case series is the emphasis on quality-of-life improvements. Patients with painful lesions reported enhanced ability to eat, speak, and chew, aligning with improvements in functional and psychosocial parameters noted in other studies.^8^ This highlights apremilast's role not only in disease control, but also in addressing the broader impact of OLP on daily living.

Our retrospective case series at a single clinical site is limited by its small population size. Notably, all patients in our case series and literature review were female, reflecting the higher incidence of OLP in women, but limiting generalizability to male patients. There are also confounding factors affecting our observations, notably the heterogeneity of treatment protocols, which restricts comparisons and definitive conclusions regarding treatment efficacy. No formal selection criteria were used prior to starting apremilast for our patients, introducing selection bias. We focused our analysis on subjective measures, increasing risk of observer bias. While the series provides compelling anecdotal evidence, larger randomized controlled trials are required to establish standardized protocols for apremilast use in OLP. Furthermore, direct comparisons between apremilast and other systemic therapies are needed to better define its place in the treatment algorithm. Moving forward, identifying predictors of treatment response will help tailor therapy and optimize patient outcomes.

## Conflicts of interest

None disclosed.
